# Self-measured blood pressure service and device use increased among Medicaid enrollees from 2018-2022

**DOI:** 10.1093/haschl/qxaf143

**Published:** 2025-07-24

**Authors:** Nathan Pauly, Jared Augenstein, Janet Williams, Kate Kirley, Emily Polk, Gregory Wozniak

**Affiliations:** Manatt Health Strategies, Chicago, IL 60606, United States; Manatt Health Strategies, New York, NY 10036, United States; American Medical Association, Improving Health Outcomes, Chicago, IL 60611, United States; American Medical Association, Improving Health Outcomes, Chicago, IL 60611, United States; Manatt Health Strategies, San Francisco, CA 94111, United States; American Medical Association, Improving Health Outcomes, Chicago, IL 60611, United States

**Keywords:** medicaid, hypertension, blood pressure, home blood pressure, SMBP, self-measured blood pressure, coverage, cardiovascular disease, home blood pressure monitoring, medicaid claims

## Abstract

**Introduction:**

When coupled with clinical supports, self-measured blood pressure (SMBP) monitoring has been shown to be effective at lowering blood pressure for people with hypertension, with positive impacts on health equity.

**Methods:**

This analysis uses nationwide Medicaid claims data to examine trends in SMBP use (ie, use of SMBP services and/or devices) at the national and state levels from 2018 to 2022 among adult Medicaid enrollees.

**Results:**

Rates of SMBP use spiked during the pandemic and subsequently decreased, although rates are still higher than those observed pre-pandemic, and utilization varied considerably by state.

**Conclusion:**

Future research should seek to improve understanding of factors driving state variation in SMBP use.

## Introduction

Hypertension, defined as blood pressure consistently at or above 130/80 mm Hg, affects almost one-third of US adults, but only about half of those adults has their hypertension under control.^1^ Hypertension is a key risk factor for cardiovascular disease, and disparities in hypertension prevalence and management are well documented, with significantly higher rates of uncontrolled hypertension among racial and ethnic minority groups.^2^

Strategies for improving blood pressure control often include self-measured blood pressure (SMBP) monitoring or the measurement of blood pressure by a patient outside of a clinical setting. When coupled with clinical supports such as patient counseling, education, telehealth, and phone support, SMBP has been shown to be both cost-effective and efficacious at lowering blood pressure and associated health risks for people with hypertension.^3^ Self-measured blood pressure with appropriate clinical supports constitute “SMBP services.” Patients can measure their blood pressure at home or in other nonclinical settings using “SMBP devices” (ie, automatic or manual devices and blood pressure cuffs), with automatic devices recommended over manual devices.^4^

Studies show SMBP can have a positive impact on health equity and can successfully be implemented in safety net settings.^5^

National organizations recommend out-of-office blood pressure measurements to confirm hypertension diagnoses and for titration of medications, combined with clinical supports, as a best practice,^4^ and health care organizations have advocated for payers to cover SMBP. State Medicaid programs have expanded coverage of SMBP in recent years, with 42 state Medicaid programs covering SMBP devices in February 2024.^6^

However, 1 study found that self-reported SMBP use (ie, use of SMBP services or devices) did not change from 2019 to 2021 across 14 states.^7^ Other forms of remote physiologic monitoring (RPM), however, have increased in use since the COVID-19 Public Health Emergency, including among Medicaid enrollees.^8^

This paper examines trends in SMBP use at the national and state levels from 2018 to 2022 among adult Medicaid enrollees, as well as characteristics of providers billing and patients using SMBP services and devices in 2022. These results provide insights to support state Medicaid agencies and policymakers considering SMBP coverage and payment policies.

## Data and methods

We utilized Transformed Medicaid Statistical Information System Analytic Files (TAF) data to investigate SMBP service and device use among Medicaid enrollees from January 2018 to December 2022. The TAF data contain Medicaid fee-for-service (FFS) claims, managed care encounters, and enrollment information from all states.

Individuals were classified as having hypertension if they had at least 1 medical claim with a hypertension-related ICD-10 diagnosis code (see Table S1). Individuals 18 years and older were included in the analysis.

We identified SMBP service and device use using Current Procedural Terminology (CPT®) codes on outpatient medical claims (see Supplementary material). Individuals were classified as SMBP recipients if they had at least 1 claim with a relevant code during the study period. We assume that individuals with claims or encounters with SMBP-related service codes (99473 or 99474) or device codes (A4660, A4670, and A4663) are using SMBP devices.

Annual rates of SMBP service and device use by state were calculated as the number of individuals with SMBP service or device claims per 1000 Medicaid enrollees with hypertension in a state.

Providers that billed at least 1 claim for an SMBP device or service in 2022 were classified as SMBP providers. We quantified the concentration of SMBP providers by calculating the number of SMBP patients and claims attributable to providers in the top 5 and 25 percentiles of SMBP service and device claim volume in 2022.

We classified whether states had SMBP coverage policies in March 2023 using data compiled in SMBP Coverage Insights: Medicaid.^6^ We assessed whether states with documented coverage policies for SMBP services or devices had higher rates of use in 2022 relative to other states.

### Data limitations

The TAF data only include information necessary for Medicaid claims adjudication, may contain errors or inaccuracies in coding or have missing data, and are subject to state-specific data quality issues.^9^ This analysis also assumes that individuals with claims or encounters that include SMBP-related service or device codes are using SMBP devices, but this may not always be the case if patients receive a device and ultimately choose not to use it. The analysis comparing rates of SMBP use in states that did/did not have documented coverage policies in place relied on publicly available data collected by the American Medical Association and may not reflect coverage policies among Medicaid managed care organizations, which can choose to cover services above and beyond those covered by a state's FFS Medicaid program.

## Results

### Trends in SMBP use

From 2018 to 2022, the number of SMBP service or device recipients increased from 9.4 to 13.5 recipients per 1000 adult Medicaid enrollees with hypertension.

During this time, SMBP use rates peaked in 2020 and declined through 2022 (see Figure S1). In 2022, 96.2% of adult Medicaid enrollees that received SMBP services or devices received automatic SMBP devices; only 2.9% received SMBP services. Among individuals receiving SMBP services, 84.9% had claims with a procedure code (99473) reflecting patient education/training and device calibration, while only 17.4% had claims with a procedure code (99474) reflecting collection of data by a provider and subsequent communication of a treatment plan (percents do not sum to 100 because some patients had both types of claims).

**Figure 1.**
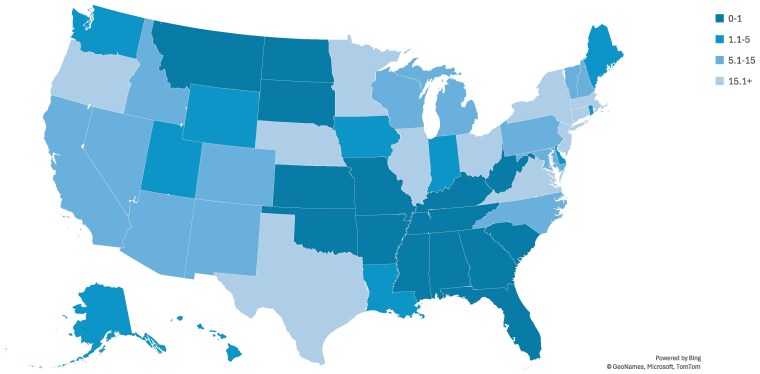
Rates of SMBP use per 1000 adult medicaid enrollees with hypertension by state, 2022. Source: T-MSIS Analytic Files (TAF) data from January 1, 2022, to December 31, 2022. Notes: Rates of SMBP service and device use were calculated as the number of distinct individuals with SMBP service or device claims per 1000 total Medicaid enrollees with hypertension in a given state in 2022. The number of individuals with SMBP service or device claims is censored from TAF data when results are under 10 individuals. This applies to the following states: Mississippi, Montana, North Dakota, South Carolina, South Dakota, and West Virginia. Assuming any number from 0 to 10 individuals receiving SMBP services or devices in these states results in a SMBP rate in the 0-1 category above. Abbreviation: SMBP, self-measured blood pressure.

Rates of SMBP use among adult Medicaid enrollees varied significantly across states. State-level rates in 2022 ranged from a low of 0 recipients in the Virgin Islands to a high of 58.4 recipients per 1000 enrollees with hypertension in DC (Figure 1). ANOVA tests were conducted to confirm rates differed significantly across states.

Self-measured blood pressure use in most states increased from 2018 to 2022, but SMBP use decreased in 11 states (data not shown).

We also compared state-level rates of SMBP service and device use among states that did and did not have coverage policies in place. We found states that had coverage policies for SMBP automated devices had on average over 2 times higher rates of SMBP automated device use relative to other states (see Table S2).

### Provider concentration (2022)

We identified 327 providers who billed SMBP service claims in 2022 for 4654 patients, and SMBP service billing was highly concentrated among a small number of providers (see Table S3). The 17 providers in the top 5 percentile of SMBP service volume accounted for over 70% of patients who received SMBP services and over 76% of claims for SMBP services.

We identified 2267 providers who billed SMBP device claims in 2022 for 144 873 patients and found SMBP device billing was also concentrated among a small group of providers, with the 114 providers in the top 5 percentile of SMBP device volume accounting for the top 68% of SMBP device patients and claims.

### Characteristics of SMBP recipients (2022)

We identified 147 930 individuals that used an SMBP service or device in 2022. Most SMBP recipients were aged 46+, although rates of SMBP use were higher among younger adults (ie, 22.9 SMBP recipients per 1000 individuals with hypertension aged 19-25, vs 12.2 SMBP recipients per 1000 individuals with hypertension aged > 65) (Table 1).

**Table 1.** Demographic and medicaid enrollment characteristics of adults that used SMBP services or devices, 2022.

Characteristics	Percent of all Medicaid-enrolled adults with hypertension	Percent of all SMBP service or device recipients	Rate of SMBP service or device use per 1000 Medicaid-enrolled adults with hypertension
**Age group (years)**			
19-25	3.0%	5.1%	22.9
26-35	8.0%	11.6%	19.6
36-45	12.9%	12.6%	13.3
46-65	46.7%	44.2%	12.8
>65	29.3%	26.5%	12.2
**Sex**			
Female	59.1%	66.9%	15.3
Male	40.9%	33.1%	11.0
Missing	0.0%	0.0%	0.0
**Race/ethnicity**			
White, non-Hispanic	38.4%	27.0%	9.5
Black, non-Hispanic	21.8%	28.4%	17.7
Asian, non-Hispanic	5.9%	6.8%	15.5
American Indian/Alaska Native, non-Hispanic	1.2%	0.6%	7.3
Hawaiian/Pacific Islander	0.8%	1.1%	17.2
Multiracial, non-Hispanic	0.4%	0.4%	11.7
Hispanic, all races	19.6%	24.0%	16.6
Other, non-Hispanic	0.8%	1.1%	16.9
Missing	11.0%	10.6%	13.0
**Managed care vs fee-for-service enrollment**			
Fee-for-service	18.7%	10.1%	7.4
Limited managed care	14.9%	15.6%	14.2
Comprehensive managed care	66.5%	74.3%	15.1
**Statistical area**			
Metropolitan	83.4%	92.3%	15.0
Micropolitan	9.3%	4.9%	7.2
Rural	7.3%	2.8%	5.2

Source: T-MSIS Analytic Files (TAF) data from January 1, 2022, to December 31, 2022.

Abbreviation: SMBP, self-measured blood pressure.

Rates of SMBP use among individuals who were Black, Asian, Hawaiian/Pacific Islander, or Hispanic were much higher than—and in some cases, nearly double—rates among individuals who were White. *χ*^2^ tests confirmed significant differences between SMBP recipients and nonrecipients with hypertension in terms of all characteristics assessed (data not shown).

## Discussion

Rates of SMBP use spiked during the pandemic and subsequently decreased, although rates are still higher than those observed pre-pandemic. This trend aligns with other telehealth services more generally.^8^

However, prior studies have found that rates of some telehealth services, like RPM (which over two-thirds of state Medicaid programs cover), increased rapidly during the COVID-19 Public Health Emergency, and rates have been sustained in recent years among Medicaid enrollees.^8^ One hypothesis for this difference is that RPM reimbursement is generally higher than SMBP reimbursement, driving more sustained RPM utilization compared to SMBP utilization.

We found that SMBP use was concentrated among older adults (46+), a population with higher rates of hypertension compared to younger adults.^1^ However, younger adults were more likely to use SMBP services or devices, potentially due to increased comfort with remote care modalities. We also found that rates of SMBP use were much higher among individuals in metropolitan areas relative to micropolitan or rural areas, which may be attributable to better access to care, innovative providers, and access to the internet.

Rates of SMBP use among most racial or ethnic minority groups—including among Black, Asian, Hawaiian/Pacific Islander, and Hispanic populations—were relatively high, compared to White populations. High rates of SMBP use among these groups are promising given SMBP's demonstrated impact on health equity.^5^ Additional SMBP adoption could further address disparities in hypertension treatment outcomes.

We observed significant variability in rates of SMBP use at the state level, indicating that Medicaid enrollees in some states could benefit from increased SMBP use. For example, southeastern states have relatively high hypertension prevalence^10^ but generally had lower SMBP use rates in 2022, underscoring potential opportunities to increase uptake.

Variation in state-level rates may be partially attributable to state-level variation in SMBP service or device coverage. We found that states with documented coverage policies in place for SMBP devices had much higher rates of device use than other states. Providers may be more willing and able to prescribe SMBP devices in states with clear coverage policies.

Conversely, data showed lower rates of SMBP service use among states with coverage policies for SMBP services, although rates of SMBP service use were generally low throughout the study period and we did not conduct statistical analyses to assess the significance of observed differences. The low rates of SMBP service use may be partially driven by providers that are prescribing SMBP devices but are unaware that they can also bill for services related to SMBP device education and calibration or data collection. It is also possible that providers are in fact rendering these services but are not submitting relevant claims and encounters, potentially due to states having unclear coverage policies, or are billing using remote patient monitoring (RPM) service codes instead of SMBP service codes.

There are several reasons why patients may have claims and encounters for SMBP services and devices even though their states do not have publicly documented coverage policies. For example, providers in states without publicly available coverage policies could be responding to informal Medicaid agency guidance or forces outside of Medicaid, such as increasing commercial coverage of SMBP services and devices. Additionally, it is possible that in some cases, Medicaid managed care plans cover SMBP services or devices, even when the state does not cover them through Medicaid FFS; a state could therefore be classified as not having SMBP coverage, while managed care enrollees may in fact have access to SMBP services and devices.

Medicaid agencies have several levers for increasing appropriate SMBP use, including (1) ensuring adequate coverage and reimbursement for SMBP services and devices, including requiring coverage and payment for SMBP services and devices in Medicaid managed care and ensuring minimal administrative burden (eg, prior authorization); (2) disseminating coverage policies to providers likely to bill these services; (3) developing technical assistance and educational resources for providers explaining the benefits of SMBP use and the services that may be reimbursed alongside SMBP devices; and (4) working with commercial payers to encourage benefit alignment.

Future research should seek to improve understanding of factors driving variation in state-level SMBP use, such as coverage policies, reimbursement rates, income and age distribution, access to the internet, and social drivers of health.

## Supplementary Material

qxaf143_Supplementary_Data
