# Supplementary material for: Self-measured blood pressure service and device use increased among Medicaid enrollees from 2018-2022
Source: Health Aff Sch. 2025 Jul 24;3(8):qxaf143. doi: 10.1093/haschl/qxaf143 (PMC12366784; doi:10.1093/haschl/qxaf143)
Supplement: qxaf143_Supplementary_Data [file qxaf143_supplementary_data.zip › Medicaid SMBP_HA Scholar Supplementary.docx]

**Supplementary Materials**

SMBP services and devices were identified using the following Current Procedural Terminology (CPT®) codes:

- 99473 and 99474 for SMBP-related services;
- A4660 for SMBP manual devices;
- A4670 for SMBP automatic devices; and
- A4663 for SMBP standalone inflatable cuffs.

Hypertension related ICD-10 diagnosis codes were identified using Clinical Classification Software from the Agency for Healthcare Research and Quality (AHRQ). These codes are listed in the table below.

**Supplementary Materials Table 1**

Caption: Hypertension-Related ICD-10 Codes

Source: Clinical Classification Software from the Agency for Healthcare Research and Quality (AHRQ)

| ICD-10 Code | Code Description |
| --- | --- |
| H35031 | Hypertensive retinopathy, right eye |
| H35032 | Hypertensive retinopathy, left eye |
| H35033 | Hypertensive retinopathy, bilateral |
| I10 | Essential (primary) hypertension |
| I110 | Hypertensive heart disease with heart failure |
| I119 | Hypertensive heart disease without heart failure |
| I120 | Hypertensive chronic kidney disease with stage 5 chronic kidney disease or end stage renal disease |
| I129 | Hypertensive chronic kidney disease with stage 1 through stage 4 chronic kidney disease, or unspecified chronic kidney disease |
| I130 | Hypertensive heart and chronic kidney disease with heart failure and stage 1 through stage 4 chronic kidney disease, or unspecified chronic kidney disease |
| I1310 | Hypertensive heart and chronic kidney disease without heart failure, with stage 1 through stage 4 chronic kidney disease, or unspecified chronic kidney disease |
| I1311 | Hypertensive heart and chronic kidney disease without heart failure, with stage 5 chronic kidney disease, or end stage renal disease |
| I132 | Hypertensive heart and chronic kidney disease with heart failure and with stage 5 chronic kidney disease, or end stage renal disease |
| I150 | Renovascular hypertension |
| I151 | Hypertension secondary to other renal disorders |
| I152 | Hypertension secondary to endocrine disorders |
| I158 | Other secondary hypertension |
| I159 | Secondary hypertension, unspecified |
| I160 | Hypertensive urgency |
| I161 | Hypertensive emergency |
| I169 | Hypertensive crisis, unspecified |
| I674 | Hypertensive encephalopathy |
| I973 | Postprocedural hypertension |
| O10011 | Pre-existing essential hypertension complicating pregnancy, first trimester |
| O10012 | Pre-existing essential hypertension complicating pregnancy, second trimester |
| O10013 | Pre-existing essential hypertension complicating pregnancy, third trimester |
| O10019 | Pre-existing essential hypertension complicating pregnancy, unspecified trimester |
| O1002 | Pre-existing essential hypertension complicating childbirth |
| O1003 | Pre-existing essential hypertension complicating the puerperium |
| O10111 | Pre-existing hypertensive heart disease complicating pregnancy, first trimester |
| O10112 | Pre-existing hypertensive heart disease complicating pregnancy, second trimester |
| O10113 | Pre-existing hypertensive heart disease complicating pregnancy, third trimester |
| O10119 | Pre-existing hypertensive heart disease complicating pregnancy, unspecified trimester |
| O1012 | Pre-existing hypertensive heart disease complicating childbirth |
| O1013 | Pre-existing hypertensive heart disease complicating the puerperium |
| O10211 | Pre-existing hypertensive chronic kidney disease complicating pregnancy, first trimester |
| O10212 | Pre-existing hypertensive chronic kidney disease complicating pregnancy, second trimester |
| O10213 | Pre-existing hypertensive chronic kidney disease complicating pregnancy, third trimester |
| O10219 | Pre-existing hypertensive chronic kidney disease complicating pregnancy, unspecified trimester |
| O1022 | Pre-existing hypertensive chronic kidney disease complicating childbirth |
| O1023 | Pre-existing hypertensive chronic kidney disease complicating the puerperium |
| O10311 | Pre-existing hypertensive heart and chronic kidney disease complicating pregnancy, first trimester |
| O10312 | Pre-existing hypertensive heart and chronic kidney disease complicating pregnancy, second trimester |
| O10313 | Pre-existing hypertensive heart and chronic kidney disease complicating pregnancy, third trimester |
| O10319 | Pre-existing hypertensive heart and chronic kidney disease complicating pregnancy, unspecified trimester |
| O1032 | Pre-existing hypertensive heart and chronic kidney disease complicating childbirth |
| O1033 | Pre-existing hypertensive heart and chronic kidney disease complicating the puerperium |
| O10411 | Pre-existing secondary hypertension complicating pregnancy, first trimester |
| O10412 | Pre-existing secondary hypertension complicating pregnancy, second trimester |
| O10413 | Pre-existing secondary hypertension complicating pregnancy, third trimester |
| O10419 | Pre-existing secondary hypertension complicating pregnancy, unspecified trimester |
| O1042 | Pre-existing secondary hypertension complicating childbirth |
| O1043 | Pre-existing secondary hypertension complicating the puerperium |
| O10911 | Unspecified pre-existing hypertension complicating pregnancy, first trimester |
| O10912 | Unspecified pre-existing hypertension complicating pregnancy, second trimester |
| O10913 | Unspecified pre-existing hypertension complicating pregnancy, third trimester |
| O10919 | Unspecified pre-existing hypertension complicating pregnancy, unspecified trimester |
| O1092 | Unspecified pre-existing hypertension complicating childbirth |
| O1093 | Unspecified pre-existing hypertension complicating the puerperium |
| O111 | Pre-existing hypertension with pre-eclampsia, first trimester |
| O112 | Pre-existing hypertension with pre-eclampsia, second trimester |
| O113 | Pre-existing hypertension with pre-eclampsia, third trimester |
| O114 | Pre-existing hypertension with pre-eclampsia, complicating childbirth |
| O115 | Pre-existing hypertension with pre-eclampsia, complicating the puerperium |
| O119 | Pre-existing hypertension with pre-eclampsia, unspecified trimester |
| O131 | Gestational [pregnancy-induced] hypertension without significant proteinuria, first trimester |
| O132 | Gestational [pregnancy-induced] hypertension without significant proteinuria, second trimester |
| O133 | Gestational [pregnancy-induced] hypertension without significant proteinuria, third trimester |
| O134 | Gestational [pregnancy-induced] hypertension without significant proteinuria, complicating childbirth |
| O135 | Gestational [pregnancy-induced] hypertension without significant proteinuria, complicating the puerperium |
| O139 | Gestational [pregnancy-induced] hypertension without significant proteinuria, unspecified trimester |
| O1400 | Mild to moderate pre-eclampsia, unspecified trimester |
| O1402 | Mild to moderate pre-eclampsia, second trimester |
| O1403 | Mild to moderate pre-eclampsia, third trimester |
| O1404 | Mild to moderate pre-eclampsia, complicating childbirth |
| O1405 | Mild to moderate pre-eclampsia, complicating the puerperium |
| O1410 | Severe pre-eclampsia, unspecified trimester |
| O1412 | Severe pre-eclampsia, second trimester |
| O1413 | Severe pre-eclampsia, third trimester |
| O1414 | Severe pre-eclampsia complicating childbirth |
| O1415 | Severe pre-eclampsia, complicating the puerperium |
| O1420 | HELLP syndrome (HELLP), unspecified trimester |
| O1422 | HELLP syndrome (HELLP), second trimester |
| O1423 | HELLP syndrome (HELLP), third trimester |
| O1424 | HELLP syndrome, complicating childbirth |
| O1425 | HELLP syndrome, complicating the puerperium |
| O1490 | Unspecified pre-eclampsia, unspecified trimester |
| O1492 | Unspecified pre-eclampsia, second trimester |
| O1493 | Unspecified pre-eclampsia, third trimester |
| O1494 | Unspecified pre-eclampsia, complicating childbirth |
| O1495 | Unspecified pre-eclampsia, complicating the puerperium |
| O1500 | Eclampsia complicating pregnancy, unspecified trimester |
| O1502 | Eclampsia complicating pregnancy, second trimester |
| O1503 | Eclampsia complicating pregnancy, third trimester |
| O151 | Eclampsia complicating labor |
| O152 | Eclampsia complicating the puerperium |
| O159 | Eclampsia, unspecified as to time period |
| O161 | Unspecified maternal hypertension, first trimester |
| O162 | Unspecified maternal hypertension, second trimester |
| O163 | Unspecified maternal hypertension, third trimester |
| O164 | Unspecified maternal hypertension, complicating childbirth |
| O165 | Unspecified maternal hypertension, complicating the puerperium |
| O169 | Unspecified maternal hypertension, unspecified trimester |

**Supplementary Materials Figure 1**

Caption: Rates of SMBP Use Per 1,000 Adult Medicaid Enrollees With Hypertension, National Trend, 2018 -2022

Source: T-MSIS Analytic Files (TAF) data from January 1, 2018, to December 31, 2022.

Notes:

- The annual rates were calculated based on the total number of individuals with SMBP service or device claims per 1,000 Medicaid enrolled adults with hypertension.
- SMBP = Self-measured blood pressure

**Supplementary Materials Table 2**

Caption: Rates of SMBP Service and Device Use per 1,000 Adult Medicaid Enrollees with Hypertension Among States That Do / Do Not Cover SMBP Services and Devices, 2022

Source: T-MSIS Analytic Files (TAF) data from January 1, 2022, to December 31, 2022.

Notes:

- States were classified as having SMBP coverage policies in place in March 2023 using data provided by the American Medical Association (AMA)
- Rates of SMBP service and device use were calculated as the number of distinct individuals with SMBP service or device claims per 1,000 total Medicaid enrollees with hypertension in a given set of states state in 2022.
- It is possible for providers to submit claims and encounters with SMBP-related procedure codes even if states do not have publicly documented SMBP coverage policies in place. This may occur for several reasons: Providers in states without publicly available coverage policies could be responding to informal Medicaid agency guidance or forces outside of Medicaid, such as increasing commercial coverage of SMBP services and devices. Additionally, it is possible that in some cases, Medicaid managed care plans cover SMBP services or devices, even when the state does not cover them through Medicaid fee-for-service; a state could therefore be classified as not having SMBP coverage, while managed care enrollees may in fact have access to SMBP services and devices.
- SMBP = Self-measured blood pressure

| **Coverage Category** | **Documented Coverage Policy** | **Number of States** | **Rate of SMBP use per 1,000 Medicaid-enrolled adults with hypertension** |
| --- | --- | --- | --- |
| **SMBP Services** | No | 30 | 0.53 SMBP service recipients per 1,000 |
|  | Yes | 21 | 0.19 SMBP service recipients per 1,000 |
| **SMBP Automatic Devices** | No | 14 | 6.18 SMBP automatic device recipients per 1,000 |
|  | Yes | 37 | 15.15 SMBP automatic device recipients per 1,000 |
| **SMBP Standalone Cuffs** | No | 19 | 0.48 SMBP standalone cuff recipients per 1,000 |
|  | Yes | 32 | 0.37 SMBP standalone cuff recipients per 1,000 |

**Supplementary Materials Table 3**

Caption: Concentration of SMBP Service and Device Billing Among Providers, 2022

Source: T-MSIS Analytic Files (TAF) data from January 1, 2022, to December 31, 2022.

Notes:

- Providers were classified as SMBP providers if they billed at least one claim for an SMBP device or service in 2022
- SMBP providers were ranked by percentile of SMBP claims billed in 2022.
- SMBP = Self-measured blood pressure

| **SMBP Service Claims** | | | |
| --- | --- | --- | --- |
| Provider SMBP Service Claim Volume Percentile | Providers N | Claims N (%) | Patients N (%) |
| 96 - 100% | 17 | 5,074 (76%) | 3,319 (71%) |
| 76 - 95% | 70 | 1,159 (17%) | 952 (20%) |
| 0 - 75% | 240 | 417 (6%) | 383 (8%) |
| Total | 327 | 6,650 (100%) | 4,654 (100%) |
| **SMBP Devices** | | | |
| Provider SMBP Device Claim Volume Percentile | Providers N | Claims N (%) | Patients N (%) |
| 96 - 100% | 114 | 102,536 (68%) | 98,449 (68%) |
| 76 - 95% | 454 | 35,326 (24%) | 34,479 (24%) |
| 0 - 75% | 1,699 | 12,144 (8%) | 11,945 (8%) |
| Total | 2,267 | 150,006 (100%) | 144,873 (100%) |
